# Retraction note: Serotonin 5-HT^7^ receptor agonist, LP-211, exacerbates Na^+^, K^+^-ATPase/Mg2^+^ ATPase imbalances in spinal cord-injured male rats

**DOI:** 10.1186/s13000-016-0563-6

**Published:** 2016-11-02

**Authors:** Abbas Norouzi-Javidan, Javad Javanbakht, Fardin Barati, Nahid Fakhraei, Fatemeh Mohammadi, Ahmad Reza Dehpour

**Affiliations:** 1 Brain and Spinal Cord Injury Research Center, Neuroscience Institute, Tehran University of Medical Sciences, Tehran, Iran; 2 Experimental Medicine Research Center, School of Medicine, Tehran University of Medical Sciences, Tehran, Iran; 3 Department of Pharmacology, School of Medicine, Tehran University of Medical Sciences, P.O. Box 13145-784, Tehran, Iran

## Retraction

The Editor-in-Chief and Publisher have retracted this article [1] because the scientific integrity of the content cannot be guaranteed. An investigation by the Publisher found it to be one of a group of articles we have identified as showing evidence suggestive of attempts to subvert the peer review and publication system to inappropriately obtain or allocate authorship. This article showed evidence of plagiarism (most notably from the articles cited [2–5]) and authorship manipulation.
